# Polymorphisms of the *plasmodium falciparum* dihydropteroate synthase gene among patients attending LEPI and ADLUCEM hospitals in the west region of Cameroon

**DOI:** 10.1016/j.parepi.2026.e00510

**Published:** 2026-05-01

**Authors:** Guela Djoukou Mba Edna, Noumedem Anangmo Christelle Nadia, Yamssi Cedric, Simeni Njonnou Sylvain Raoul, Ngongang Ouankou Christian, Tchuenkam Kom Pacome, Ngouyamsa Nsapkain Aboubakar Sidiki, Tientcheu Noutong Jemimah Sandra, Toumko Towa Merveille, Guemegne Anaiss Patricia Line, Kana Tsague Yval, Haibo Hu, Simeon Pierre Choukem

**Affiliations:** a Department of Microbiology, Haematology and Immunology, Faculty of Medicine and Pharmaceutical Sciences, University of Dschang, Cameroon; b Laboratory of Tropical and Emerging Infectious Diseases, Dschang, Cameroon; c Department of Biomedical Sciences, University of Bamenda, Bambili, Cameroon; d Department of Internal Medecine and Specialties, Faculty of Medicine and Pharmaceutical Sciences, University of Dschang, Cameroon; e Research Unit of Microbiology and Antimicrobial Substances, Department of Biochemistry, Faculty of Science, University of Dschang, Dschang, Cameroon; f National Engineering Research Center for Modernization of Traditional Chinese Medicine-Hakka Medical Resources Branch, School of Pharmacy, Gannan Medical University, Ganzhou, China; g Health and Human development (2HD) Research Network, Douala, Cameroon

**Keywords:** Malaria, Drug resistance, *Pfdhps*, A437G, A581G, Cameroon

## Abstract

**Background:**

Cameroon bears a high malaria burden, and sulfadoxine–pyrimethamine (SP) remains widely used for intermittent preventive treatment in pregnancy despite the adoption of artemisinin-based combination therapies. Continued programmatic use of SP under sustained malaria transmission exerts selective pressure for resistant *Plasmodium falciparum* strains. This study investigated the molecular epidemiology of resistance-associated mutations in the *P. falciparum* dihydropteroate synthase (*Pfdhps*) gene in two health facilities in the Mbouda Health District, Cameroon.

**Methodology:**

Blood samples were collected from consenting patients, and tested using a rapid diagnostic test (RDT) and thick blood smear to detect *P. falciparum* and determine parasite density. Positive samples were spotted onto Whatman filter paper for molecular analysis. Parasite DNA was extracted using the Chelex 100 method. The Pfdhps gene was amplified by semi-nested PCR, and restriction fragment length polymorphism (RFLP) analysis was performed using *AvaII* and *BstUI* to detect the A437G and A581G mutations, respectively.

**Results:**

Among the 286 samples (198 women and 88 men aged 29 ± 20 years) were collected, *P. falciparum* was present in 87 (30.41%). Male participants had a slightly higher mean parasite density (2343 ± 1240 parasites/μL) compared to females (2283 ± 1483 parasites/μL). Education level was also significantly associated with positivity (*p* = 0.001), with the highest prevalence among participants with primary education only (41.66%) and the lowest among those with no formal schooling (10.34%). The frequency of the A581G mutant allele was 67.60%, while that of A437G was 45.07%. The wild-type allele frequencies were 48.87% for A437G and 22.53% for A581G. Mixed alleles were observed only for A437G (1.40%).

**Conclusion:**

The high frequency of *Pfdhps* mutations suggests substantial sulfadoxine resistance pressure in the study area. However, as *dhfr* mutations associated with pyrimethamine resistance were not assessed, overall sulfadoxine–pyrimethamine efficacy cannot be conclusively determined.

## 1 Introduction

Malaria is a parasitic disease caused by protozoa of the genus *Plasmodium*. According to the World Health Organization (WHO)’s latest World malaria report 2025, malaria remains a serious global health challenge, with an estimated 282 million cases and 610,000 deaths in 2024 – roughly 9 million more cases than the previous year (World Health Organization, 2025). Malaria is present in more than one hundred countries, with a disproportionate burden in low-income tropical regions of Africa, Asia, and Latin America (Gimaro and Risenga, 2025). Sub-Saharan Africa alone accounts for roughly 94% of all global malaria cases (Sibomana et al., 2025). According to the WHO's annual malaria report, the COVID-19 pandemic has contributed to a significant increase in malaria incidence and mortality rates (Liu et al., 2022). The report highlights that the total number of malaria cases worldwide in 2022 was considerably higher than before the pandemic in 2019. After a gradual decline from 243 million cases in 2000 to 233 million in 2019, the trend reversed: there were 11 million additional cases in 2020, no change in 2021, and a further increase of 5 million cases in 2022, reaching approximately 249 million cases overall. Over the past decades, *Plasmodium* parasites have gradually acquired resistance to several antimalarial drugs, while mosquito vectors have shown declining susceptibility to conventional control measures (Nikiema et al., 2025).

In Cameroon, malaria continues to be endemic and represents a leading cause of morbidity and mortality, accounting for an estimated 11,600 deaths in 2024 (Bizimana Rukundo, 2026). Although artemisinin-based combination therapies (ACTs) are recommended for the treatment of uncomplicated malaria, sulfadoxine–pyrimethamine (SP) remains a cornerstone of malaria prevention strategies (Bizimana Rukundo, 2026). In particular, SP is used for intermittent preventive treatment in pregnancy (IPTp) as part of national malaria control guidelines and contributes significantly to reducing malaria-associated maternal anemia, placental malaria, low birth weight, and neonatal mortality (Briand et al., 2007). In some settings, SP has also been evaluated for intermittent preventive treatment in infants (IPTi), further underscoring its continued public health relevance.

The sustained programmatic use of SP in high-transmission settings exerts selective pressure on Plasmodium falciparum populations, facilitating the emergence and spread of resistant strains. Resistance to SP is mediated by point mutations in genes encoding enzymes of the folate biosynthesis pathway, notably dihydrofolate reductase (*dhfr*), associated with pyrimethamine resistance, and dihydropteroate synthase (*dhps*), which confers resistance to sulfadoxine (Rasmussen et al., 2022). Accumulation of specific *dhps* mutations, particularly when combined with dhfr mutations, has been associated with reduced effectiveness of SP, including compromised IPTp outcomes.

One of the main obstacles to malaria control is the emergence and spread of resistance to antimalarial drugs (Rasmussen et al., 2022). Agents such as sulfadoxine–pyrimethamine (SP), once highly effective for chemoprevention and treatment of malaria, are now losing efficacy. This resistance is primarily linked to point mutations in key parasite genes, notably *Pfdhps*, which is involved in SP resistance (Yan et al., 2021). Such mutations alter the structure of the encoded enzyme, reducing drug-binding capacity and ultimately therapeutic effectiveness (Conrad et al., 2023). This situation poses a major public health challenge, underscoring the importance of continuous molecular surveillance of *P. falciparum* genetic variations to guide treatment and prevention strategies. Ultimately, this threatens malaria control and elimination efforts across the African continent (Yan et al., 2021).

Monitoring molecular markers of SP resistance is therefore of major public health importance in Cameroon, where SP continues to be widely deployed for preventive interventions. Surveillance of resistance-associated polymorphisms provides early warning signals of declining drug effectiveness and supports evidence-based decision-making for malaria control policies (Kagoro et al., 2022). In this context, the *Pfdhps* gene represents a critical target for molecular surveillance, as sulfadoxine activity is essential for the prophylactic efficacy of SP.

The objective of this study was to investigate the molecular epidemiology of *Plasmodium falciparum* dihydropteroate synthase (*Pfdhps*) gene polymorphisms in patients attending two health facilities within the Mbouda Health District, Cameroon, and to explore potential associations between parasite prevalence, parasite density, and *Pfdhps* mutations with socio-demographic characteristics. This approach aims to provide insights into factors influencing the distribution of resistance-associated polymorphisms and to monitor the potential emergence of antimalarial drug resistance.

## 2 Materials and methods

### 2.1 Study design and study site

**Fig. 1:**
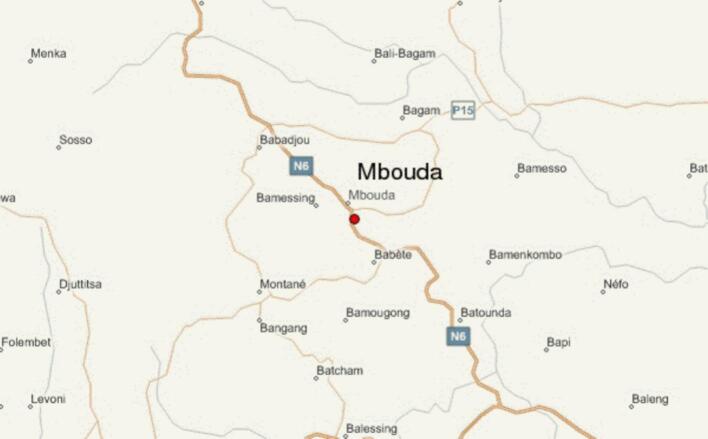
Map of the city of Mbouda (Zeitlyn, 2024).

**Fig. 2:**
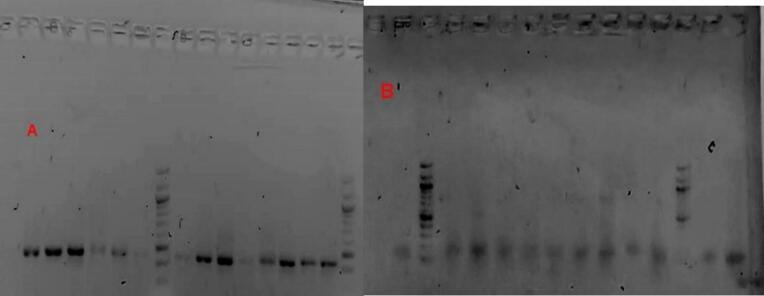
Gel electrophoresis image after Amplification of the *Pfdhps* Gene (Bands at 444 bp (A) and 161 bp (B)).

This was a cross-sectional, analytical study conducted at LEPI and AD LUCEM hospitals in Mbouda, located in the West Region of Cameroon. Mbouda lies near Mount Cameroon, covering an area of 437 km^2^ with a tropical climate and an average annual rainfall of 1964 mm. The town is situated at an altitude of 1396 m and has an estimated population of 140,000 (Lontsi et al., 2025), as shown in Fig. 1. (See Fig. 2.)

### 2.2 Inclusion and exclusion criteria

All patients attending AD LUCEM and LEPI hospitals in Mbouda for malaria diagnosis, regardless of age or sex, were eligible for inclusion upon providing informed consent or assent. Patients with coagulation disorders such as hemophilia were excluded from the study purely for ethical and safety reasons related to blood sampling.

### 2.3 Sample size determination

The minimum sample size was calculated using the Lorenz formula (de Souza et al., 2023):

$$N=\frac{P\left(1-P\right){\left(Z\right)}^{2}}{d^{2}}$$

where Z = 1.96Z = 1.96 represents a 95% confidence interval,

d = 0.05d = 0.05 is the margin of error, and *P* = 12% is the malaria prevalence in the South-west Region of Cameroon (Nyasa et al., 2021).

This calculation resulted in a minimum sample size of 162 participants.

### 2.4 Sample collection and microscopic analysis

Blood samples were collected from patients into EDTA tubes and spotted onto Whatman filter paper for molecular analysis, following the method described by Yamagata et al. (Yamagata et al., 2021). Prior to sample collection, malaria infection was initially screened using a Rapid Diagnostic Test (RDT) according to the manufacturer's instructions. Briefly, a small volume of capillary blood was applied to the test cassette, followed by the addition of the assay buffer. The results were read after 15–20 min. The RDT used in this study detects *Plasmodium falciparum* histidine-rich protein 2 (HRP2) antigen. For microscopic examination, a 5 μL drop of blood was spread on a slide, air-dried, and stained with 1:10 diluted Giemsa solution for 15 min (Sorontou, 2021). Slides were then examined under a microscope, and parasitaemia was calculated using the following formula:

$$\text{Parasitemia}=\frac{\text{Number of parasites}\;\left(\text{counted}\right)\;X\;8000\mathrm{GB}\;\mathrm{per}\;\text{microliter of blood}}{200\mathrm{GB}\;\left(\text{counted}\right)}$$

### 2.5 Molecular analysis

#### 2.5.1 DNA extraction

DNA extraction was performed using the Chelex 100 method as described by Biesen et al. *(*Van Biesen et al., *2025**)*. Briefly, a sample of Whatman filter paper containing P. falciparum-positive blood was placed in a tube containing 0.5% Tween and incubated at 4 °C for 16 h. After removing the Tween, 1 mL of phosphate-buffered saline (PBS) was added to each tube and incubated at 4 °C for 15 min. Once the PBS was removed, a 20% Chelex preparation was added to each tube. The tubes were shaken and then heated in a water bath for 15 min, repeated three times. Following centrifugation and extraction of the supernatant twice, DNA was obtained, aliquoted into a tube, and stored at −20 °C.

#### 2.5.2 Amplification of the *Pfdhps* gene

The *Pfdhps* gene was amplified using a nested PCR protocol as described by Martín et al. (Martín Ramírez et al., 2025). A total of 87 *P. falciparum*-positive samples were analysed. For the first amplification (Nest 1), 10 mM primers were prepared, and a working solution was made by mixing the master mix, primers, and nuclease-free water. Each PCR tube received 9.2 μL of this mixture and 0.8 μL of extracted DNA, then placed in a thermocycler with the programmed conditions shown in Table 1. For the second amplification (Nest 2), the reaction mix was prepared under a hood on ice, similar to Nest 1, with the nuclease-free water volume increased to 4.2 μL. Each PCR tube contained 9.6 μL of the mix and 0.4 μL of the Nest 1 amplicon. Tubes were sealed and run in the thermocycler using the appropriate program. The Nest 2 reaction mixture included 5 μL of master mix, 0.2 μL of each primer, and 4.2 μL of nuclease-free water, allowing for a more specific second amplification step.

**Table 1:** Amplification of *Pfdhps* gene.

Gene	PCR	Primers Sequences	Mutations	Amplification condition	Size in base pair(bp) fragment
*Pfdhps*	Nest I	R:5’ AACCTAAACGCTGTTCAA 3′ R/: 5’ AATTGTGTGATTTGTCCACAA3’		Pd: 94 °C(3mins), D: 94 °C(30s):, H:45 °C(30s) E:72 °C(1mins), Ef:72 °C(3mins), Td: 4 °C(+∞)	711
	Nest II	K:5’TGCTAGTGTTATAGATATAGGATGAGCATC 3′ K/: 5’CTATAACGAGGTATTGCATTTAATGCAAGAA3’	A437G	Pd: 94 °C(3mins), D: 94 °C(1mins):, H:45 °C(1mins) E:72 °C(2mins), Ef:72 °C(10mins), Td: 4 °C(+∞)	444
		L:5’ATAGGATACTATTTGATATTGGACCAGGATTCG 3′ L/: 5’TATTACAACATTTTGATCATTCGCAACCGG3’	A581G	Pd: 94 °C(3mins), D: 94 °C(1mins):, H:50 °C(1mins) E:72 °C(2mins), Ef:72 °C(10mins), Td: 4 °C(+∞)	161

**Legend:** Pd: Pre-denaturation, D: Denaturation, H: Hybridization, E: Elongation, Ef: Final elongation, Td: Hold time (or Stop time).

#### 2.5.3 Agarose gel electrophoresis and visualization

Detection of the *Pfdhps* gene was performed by electrophoresis on a 1.3% agarose gel, as described by Issa et al., (Issa et al., 2022). Briefly, the gel was placed in an electrophoresis tank filled with 0.5× TBE buffer. Samples (8 μL) were loaded into the wells, and electrophoresis was run at 150 V for 30 min. The gel was then stained with SafeView and visualized under a transilluminator.

#### 2.5.4 Restriction fragment length polymorphism (RFLP) of the Pfdhps gene

Restriction enzymes *AvaII* (for A437G) and *BstUI* (for A581G) were used as described by Ali et al., (Ali et al., 2022). Briefly, a 6.2 μL reaction mix containing water, buffer, and enzyme was prepared, followed by the addition of 3.8 μL of Nest 2 amplicon, and incubated at 37 °C for 60 min. Digestion products were visualized by agarose gel electrophoresis as described above.

### 2.6 Ethical considerations and patient consent

Ethical approval was obtained from the Regional Ethics Committee (CRERSH-West) under registration number N°425/23/03/2025/CE/CRERSH-OU/VP. Informed consent or assent was obtained from all participants prior to enrolment.

### 2.7 Statistical analysis

Data were analysed using the Statistical Package for Social Sciences version 26.0. frequencies were compared using the Chi-square test, and multivariate logistic regression was applied to identify risk factors. Results were considered statistically significant at *p* < 0.05.

## 3 Results

### 3.1 Prevalence of *Plasmodium falciparum* in two health facilities

After analyses of 286 samples 87 were found to be positives for *P. falciparum* hence the overall prevalence was 30.41%, with 34.96% at AD LUCEM and 28.33% at LEPI.

#### 3.1.1 Prevalence of *P. falciparum* according to socio-demographic characteristics

Table 2 presents the prevalence of *P. falciparum* by sex, age, marital status, and education level. No significant association was observed with sex (*p* = 0.578) or marital status (*p* = 0.101). However, age group showed a statistically significant difference (*p* = 0.001), with the highest positivity rate observed in individuals aged 5–35 years (31.84%). Education level was also significantly associated with positivity (p = 0.001), with the highest prevalence among participants with primary education only (41.66%) and the lowest among those with no formal schooling (10.34%).

**Table 2:** Prevalence of *P.falciparum* according to socio-demographic characteristics.

Variables	Number diagnosed	Number positives	Prevalence %	$x^{2}$	*p*-value
Sex				0.39	0.56
Male	88	29	32.95		
Female	198	58	29.29		
Age group				15.55	0.001
[0–5[	44	11	25.00		
[5–35[	157	50	31.84		
≥35	85	26	30.58		
Marital status				2.69	0.10
Single	150	52	34.66		
Married	136	35	25.73		
Education level				15.55	**0.001**
Primary level	84	35	41.66		
Secondary level	120	40	33.33		
Higher education	53	09	16.98		
Illiterate	29	03	10.34		

### 3.2 Parasite density according to socio-demographic characteristics

Table 3 presents parasite density stratified by socio-demographic characteristics. Male participants had a slightly higher mean parasite density (2343 ± 1240 parasites/μL) compared to females (2283 ± 1483 parasites/μL). Regarding age groups, individuals aged 5–35 years (2340 ± 1054 parasites/μL) and those ≥35 years (2077 ± 980 parasites/μL) exhibited the highest parasite loads. Analysis by education level showed that illiterate participants (2698 ± 849 parasites/μL) and those with higher education (3039 ± 2572 parasites/μL) had higher parasite densities compared to participants with primary education (2127 ± 990 parasites/μL) and secondary education (2263 ± 1413 parasites/μL).

**Table 3:** Parasite density according to socio-demographic characteristics.

Variables	Mean ± Standard Deviation
Sex	
Male	2343 ± 1240
Female	2283 ± 1483
Age group	
[0–5[	2077 ± 980
[5–35[	2340 ± 1054
≥35	2232 ± 789
Education level	
Primary level	2127 ± 990
Secondary level	2263 ± 1413
Higher education	3039 ± 2572
Illiterates	2698 ± 849

### 3.3 Frequency of the Pfdhps resistance gene amplification success

The gel electrophoresis image of the *Pfdhps* resistance gene. The presence of bands at 444 base pairs (bp) (A) and 161 bp (B) indicates the presence of this gene. Out of the 87 *Plasmodium falciparum*-positive samples analysed, 71 tested positive for the Pfdhps gene, resulting in an overall success of 81.60% in the health facilities of the Mbouda Health District. Failure in the detection of this gene is more likely attributable to technical issues such as primer design, PCR conditions, or DNA quality rather than true gene absence.

### 3.4 Allelic frequencies of A437G and A581G mutations in the Pfdhps gene

**Fig. 3:**
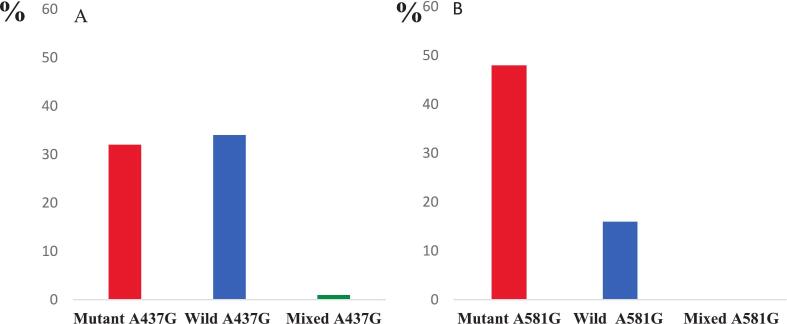
Allelic frequencies of the *Pfdhps* gene mutations (mutants A437G (A) and A581G (B)).

Gel electrophoresis images of A437G (A) and A581G (B) mutations from two health facilities in the Mbouda Health District are provided in Supplementary File S1.The presence of bands at 404 base pairs (bp) (A) and 131 and/or 105 bp (B) corresponds to the A437G and A581G mutations, respectively. Out of the 71 Pfdhps-positive samples, 32 carried the A437G mutation and 48 carried the A581G mutation. The A581G mutation was more frequent (67.60%) than A437G (45.07%). Wild-type alleles and mixed genotypes were more common for A437G than for A581G, as illustrated in Fig. 3.

#### 3.4.1 Allelic frequencies of the *Pfdhps* gene mutations A437G and A581G according to socio-demographic characteristics

Table 4 presents the association between socio-demographic variables and *Pfdhps* mutations (A437G and A581G). No statistically significant associations were observed. For sex and age group, the distribution of mutations did not differ significantly, despite slight variations in proportions. The frequency of the A437G mutation by age was relatively high but not statistically significant (*p* = 0.128). For A581G, the highest mutation rates were observed among participants with no formal education and primary education, although educational level was not significantly associated with either mutation. Overall, the mutations appeared evenly distributed across all socio-demographic groups.

**Table 4:** Allelic Frequencies of the *Pfdhps* A437G and A581G gene mutations according to socio-demographic characteristics.

Variables	Mutations A437			$x^{2}$	p-value	Mutations A581G			$x^{2}$	p-value
Sex	**Mutants (%)**	**Wild type(%)**	**Mixed (%)**	3.85	0.28	**Mutants (%)**	**Wild type (%)**	**Mixed (%)**	1.08	0.58
Male	09(40.90)	10(45.45)	01(4.45)			16(72.72)	04(18.18)	00(00)		
Female	23(46.93)	24(48.97)	00(00)			33(67.34)	12(14.48)	00(00)		
Age group				24.89	0.13				12.92	0.38
[0–5[	03(37.50)	04(50)	01(12.50)			07(87.50)	01(12.50)	00(00)		
[5–35[	07(70)	03(30)	00(00)			08(80.00)	00(00)	00(00)		
≥35	04(57.14)	03(42.85)	00(00)			07(100)	00(00)	00(00)		
Education level					0.89				9.80	0.13
Primary level	14(51.85)	12(44.44)	01(3.70)	4.32		23(85.18)	02(07.40)	00(00%)		
Secondary level	15(46.87)	15(46.87)	00(00)			19(59.37)	11(34.30)	00(00)		
Higher education	02(22.22)	05(55.55)	00(00)			04(44.44)	03(33.33)	00(00)		
Illiterates	01(33.3)	nnn(66.66)	00(00)			nn(100)	00(00)	00(00)		

#### 3.4.2 Factors associated with A437G and A581G mutations according to socio-demographic characteristics

Table 5 presents the risk factors for *Pfdhps* A437G and A581G mutations according to sex, age, and education level. No statistically significant associations were observed for either mutation. For A437G, the odds ratios across all variables were close to 1, indicating no clear trend. For A581G, the highest odds ratio was observed in participants with primary education (OR = 4.845), although this was not statistically significant (*p* = 0.155). The wide confidence intervals reflect the small sizes of some subgroups. Overall, mutation prevalence did not differ significantly by sex, age, or education level, and no socio-demographic factor was significantly associated with either mutation.

**Table 5:** Risks factors Associated with A437G and A581G mutations according to socio-demographic characteristics.

Variables	A437			Odds-ratio	95% Confidence Intervals	p-value	A581G			Odds-ratio	95% Confidence Intervals	p-value
Sex	**Mutants**	**Wild type**	**Mixed**	0.52	[0.15–1.85]	0.31	**Mutants**	**Wild type**	**Mixed**			
Male	09	10	01				16	04	00	0.63	[0.15–2.67]	0.53
Female	23	24	00				33	12	00			
Age group												
[0–5]	03	04	01	1.29	[0.04–42.10]	0.89	07	01	00			
[5–35]	21	17	00	0.90	[0.03–32.45]	0.95	26	06	00			
≥35	08	13	00	1.14	[0.38–3.38]	0.82	16	04	00			
Education level												
Primary level	14	12	01	1.37	[0.19–9.58]	0.75	23	02	00	4.85	[0.55–42.60]	0.16
Secondary level	15	15	00	1.99	[0.31–12.89]	0.47	19	11	00	1.08	[0.19–6.08]	0.93
Higher education	02	05	00				04	03	00			
Illiterates	01	02	00	0.95	[0.04–20.47]	0.97	03	00	00			

## 4 Discussion

This study provides updated epidemiological and molecular evidence on malaria transmission and antifolate resistance in the Mbouda Health District, western Cameroon. Our findings reveal a persistently high malaria burden and a worrying prevalence of resistance-associated mutations in the *Pfdhps* gene, highlighting ongoing selective pressure from sulfadoxine-pyrimethamine (SP) use in this region.

The overall malaria prevalence of 30.41% observed in this study confirms that malaria remains a major public health problem in the Mbouda Health District. This finding is consistent with previous reports from similar ecological zones in Cameroon. Chiabi et al. (Chiabi et al., 2024) reported a prevalence of 24.4%, while Mbacham et al. (Worti et al., 2025), documented similarly high transmission levels in western Cameroon. These authors attributed persistent malaria transmission to favorable climatic conditions, prolonged rainy seasons, and inadequate use of insecticide-treated nets. However, the prevalence observed in our study is higher than that reported by Chiabi et al., (Chiabi et al., 2024) suggesting a possible increase in transmission intensity over time or a decline in the effectiveness of control interventions. This difference may reflect population growth, urban expansion without adequate sanitation, and evolving vector ecology in the Mbouda Health District.

We observed a higher malaria prevalence at Adlucem (34.96%) compared to LEPI (28.33%). This finding aligns with the observations of Martin et al. (Martin and F. spécialisée en Développement, 2026), who demonstrated that population density, housing quality, and environmental sanitation strongly influence malaria transmission dynamics. Our results further support their conclusion by showing that areas with more precarious living conditions tend to sustain higher transmission. Nevertheless, the prevalence at LEPI remains substantial, indicating that even in areas with relatively better awareness and prevention efforts, malaria transmission persists. This suggests that current control strategies may be insufficient to interrupt transmission in high-burden settings.

In this study, men exhibited a higher mean parasite density (2343 trophozoites/μL) than women (2283 trophozoites/μL). This observation is in agreement with reports by Njiwale et al. (Njiwale et al., 2024) and Samake et al. (Samaké, 2021), who found that men are more frequently exposed to mosquito bites due to nighttime outdoor activities and occupational behavior.

Similarly, Gualdimo et al. (Gualdino, 2026), reported that men are less likely to seek early treatment and are less consistent in using preventive measures, which may contribute to higher parasite loads. Our findings therefore reinforce the growing evidence that gender-specific behavioral patterns play an important role in malaria transmission and disease burden.

The frequency of the A437G mutation (45.07%) observed in this study is comparable to values reported by Grandesso et al. (Grandesso et al., 2024) in Central and West Africa, confirming the widespread circulation of this mutation across the region. More importantly, the A581G mutation was detected at a very high frequency (67.60%), which is markedly higher than the 50% reported by Mbacham et al. (Worti et al., 2025), in earlier studies from Cameroon. This increase suggests a rapid expansion of highly resistant parasite strains in the Mbouda Health District. The mutation frequency observed in our study is substantially higher than that reported by Abubakar et al., (Abubakar et al., 2024) suggesting a more intense or prolonged selective pressure in western Cameroon. This difference may be explained by continued SP use for intermittent preventive treatment in pregnancy (IPTp) and frequent self-medication in the community. Suttipath et al. (Srisutham et al., 2022) demonstrated that *pfdhfr* and *pfdhps* mutations persist even after SP withdrawal, while Abubakar et al. (Abubakar et al., 2024) reported a prevalence of 42.9% in Nigeria.

Furthermore, our observation that A581G was more prevalent among women and rural populations supports the findings of Grandesso et al. (Grandesso et al., 2024), who linked this mutation to SP exposure during pregnancy and rural transmission settings. These results strongly suggest that IPTp-SP may be contributing to the selection and maintenance of highly resistant parasite populations.

While insecticide-treated bed nets remain a cornerstone of malaria prevention, our study found no statistically significant association between bed net ownership and mutation prevalence. This finding contrasts with Dahoui et al. (Dahoui, 2023), who reported a strong protective effect of consistent bed net use. The discrepancy may be explained by inconsistent or improper use of nets in our study population, suggesting that ownership alone is not a reliable indicator of protection.

Taken together, our findings provide new molecular evidence of the rapid expansion of *Pfdhps* A581G mutations in western Cameroon. Compared with earlier studies, our data suggest a worsening resistance profile, which may compromise the long-term effectiveness of SP for both treatment and prevention. These results underscore the urgent need for continuous molecular surveillance, stricter regulation of antimalarial drug use, and evaluation of alternative strategies for intermittent preventive treatment in pregnancy.

### 4.1 Study limitations

This study has some limitations that should be acknowledged. First, the study focused only on the *Pfdhps* gene, while other molecular markers associated with sulfadoxine–pyrimethamine resistance, such as mutations in the *Pfdhfr* gene, were not investigated. Furthermore, sequencing was not performed to confirm all detected mutations, which may limit the ability to identify additional or novel polymorphisms. Despite these limitations, the study provides valuable baseline data on the distribution of *Pfdhps* resistance-associated polymorphisms in the study area.

## 5 Conclusion

This study demonstrates a persistently high malaria prevalence (30.41%) in the Mbouda Health District, with parasite density influenced by socio-demographic factors. The high frequencies of the *Pfdhps* A437G and A581G mutations indicate ongoing and possibly intensifying antifolate resistance among circulating *Plasmodium falciparum* strains in this region. These findings highlight the urgent need for continuous molecular surveillance and the implementation of locally adapted malaria control strategies to limit the spread of drug-resistant parasites and preserve the effectiveness of current prevention and treatment policies. However, as *dhfr* mutations associated with pyrimethamine resistance were not assessed, overall sulfadoxine–pyrimethamine efficacy cannot be conclusively determined.

## CRediT authorship contribution statement

**Guela Djoukou Mba Edna:** Writing – original draft, Investigation. **Noumedem Anangmo Christelle Nadia:** Supervision. **Yamssi Cedric:** Methodology, Conceptualization. **Simeni Njonnou Sylvain Raoul:** Writing – original draft. **Ngongang Ouankou Christian:** Writing – original draft. **Tchuenkam Kom Pacome:** Investigation. **Ngouyamsa Nsapkain Aboubakar Sidiki:** Writing – original draft, Investigation. **Tientcheu Noutong Jemimah Sandra:** Investigation. **Toumko Towa Merveille:** Investigation. **Guemegne Anaiss Patricia Line:** Investigation. **Kana Tsague Yval:** Investigation. **Haibo Hu:** Writing – review & editing, Writing – original draft, Funding acquisition. **Simeon Pierre Choukem:** Writing – review & editing, Supervision.

## Funding

The study received no funding from any source or organization.

## Declaration of competing interest

The authors declare that they have no known competing financial interests or personal relationships that could have appeared to influence the work reported in this paper.

## Data Availability

All data generated and analysed are included in this research article.
