# Supplementary material for: Polymorphisms of the plasmodium falciparum dihydropteroate synthase gene among patients attending LEPI and ADLUCEM hospitals in the west region of Cameroon
Source: Parasite Epidemiol Control. 2026 May 1;33:e00510. doi: 10.1016/j.parepi.2026.e00510 (PMC13158770; doi:10.1016/j.parepi.2026.e00510)
Supplement: Supplementary file 1 — Supplementary material [file mmc1.docx]

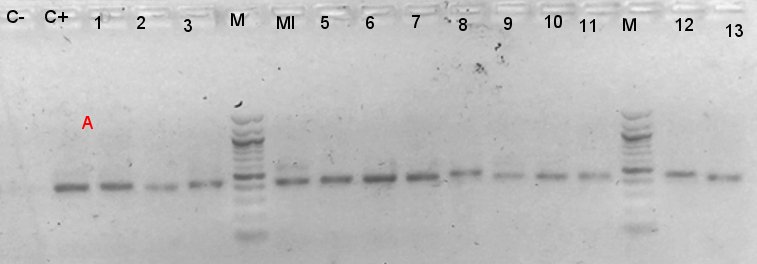

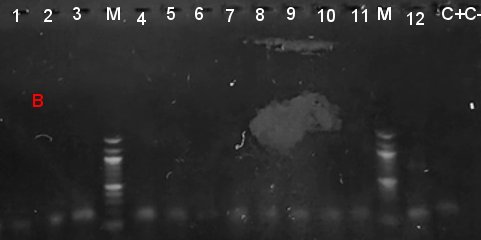


**Legend**

**C^−^ : Negative control**

**C^+^ : Positive control**

**Mi : Mixed**

**M** : **Molecular weight marker**

**S1:** Gel picture of A437 (A) and A581G (B) Mutations
